# Toward terminological clarity in digital biomarker research

**DOI:** 10.3389/fdgth.2026.1796008

**Published:** 2026-03-13

**Authors:** Madhur Mangalam, Ken Kiyono

**Affiliations:** 1 Department of Biomechanics, University of Nebraska at Omaha, Omaha, NE, United States; 2 Graduate School of Engineering Science, University of Osaka, Toyonaka, Osaka, Japan

**Keywords:** behavioral phenotypes, biological measurement, clinical translation, digital biomarker, mechanistic criteria comparison

## Abstract

Digital biomarker research has generated thousands of publications demonstrating associations between sensor-derived measures and clinical conditions, yet clinical adoption remains negligible. We identify a foundational problem: the field lacks consensus on what constitutes a digital biomarker, applying identical terminology to direct physiological measurement (continuous glucose monitoring), algorithmic prediction of biological substrates (voice analysis for dopaminergic function), and purely behavioral correlates (GPS mobility and depression scores). This terminological ambiguity obscures validation requirements and prevents evidence synthesis. We argue that the “bio-” in “digital biomarker” refers to a property of the measurement itself—the marker must be derived from biology, not merely predictive of biological or clinical outcomes. Under this restrictive definition, behavioral correlates without demonstrated biological grounding, however statistically robust or clinically useful, should be designated as digital phenotypes or digital health indicators rather than biomarkers. This distinction clarifies validation pathways: biologically derived markers require technical accuracy validation against established biological reference standards; candidate biomarkers inferred from behavior require biological criterion validation before that status is claimed; behavioral indicators require outcome prediction validation. We demonstrate how this framework resolves current validation confusion and accelerates translation by aligning evidence standards with measurement types.

## The paradox of abundance without adoption

Consider what should be a success story. A smartphone application uses the device’s motion sensors to analyze how someone walks across their living room—the rhythm of their footfalls, the variability in stride timing, the subtle asymmetries in left–right patterns. Machine learning algorithms detect patterns invisible to clinical observation. The app distinguishes people with Parkinson’s disease from healthy controls with 85% accuracy. The finding is statistically robust, published in a respected journal, and covered by scientific media. It also arrives in a field where the very label “digital biomarker” has expanded well beyond direct biological measurement. This expansive framing is reinforced by influential reviews that emphasize convergence between digital health technologies and biomarkers, often without clearly distinguishing between biological measurement and behavioral correlation (1).

Years later, no neurologist uses this approach for diagnosis, and no clinical care pathway incorporates it for monitoring or prevention.

Now multiply this story by hundreds. Smartphone keystroke patterns predicting depression (2, 3). GPS mobility correlating with psychotic symptoms (4, 5). Social media activity tracking manic episodes (6, 7). Wearable heart rate variability monitoring stress (8, 9). Screen time associated with anxiety (10, 11). Each study shows statistically significant associations. Each promises clinical utility. Few deliver.

The digital biomarker field presents a paradox: explosive research growth paired with negligible clinical adoption. We count publications in thousands, commercial investment in billions, yet evidence-based clinical guidelines rarely incorporate these measures. The gap between research enthusiasm and clinical reality continues widening.

Standard explanations blame technical challenges (12). Sensors are not accurate enough (13–15). Algorithms do not generalize (16–18). Privacy concerns limit data collection (19, 20). Regulatory pathways remain unclear (21, 22). These obstacles are real, but they may not be fundamental. We propose the translational failure runs deeper—to confusion about what we are actually measuring.

## When the same word means different things

The term “digital biomarker” now encompasses such breadth that two studies using identical terminology may be investigating categorically different phenomena. One measures interstitial glucose concentration via enzymatic reaction (23, 24). Another reports associations between smartphone app usage and self-reported depression scores (25–27). In the broader digital health literature, both kinds of measures are frequently discussed under the umbrella of “digital biomarkers” and are often considered alongside one another in review articles (21, 22). Yet they measure different types of variables, require different validation approaches, and merit different regulatory consideration. Indeed, this ambiguity is increasingly acknowledged within the field itself, with recent work explicitly questioning how much biological grounding is required for a measure to qualify as a digital biomarker (28).

This would matter less if the distinctions were merely semantic. But terminology shapes how research is designed, evaluated, and translated. When validation standards appropriate for one measurement type are misapplied to another, we get studies that appear rigorous while missing the essential evidence their claims require. When meta-analyses pool incompatible measures, we get systematic reviews that obscure rather than clarify. When regulatory agencies encounter measures that do not fit existing frameworks, translation stalls regardless of scientific quality.

The confusion stems from an unresolved question lurking beneath definitional debates: what makes a biomarker biological? This question proves less obvious than it first appears. Prior scholarship has explicitly raised these definitional concerns. Mulinari (29, 30) has argued for a restrictive definition in which “digital biomarker” is reserved for measures biologically derived at the level of measurement—a position broadly consistent with how “biomarker” is used across the non-digital field and with the FDA–NIH Working Group definition (31). Our contribution builds on and extends this line of argument by operationalizing the definitional distinction into a three-category validation framework and demonstrating its practical consequences for study design, evidence synthesis, and clinical translation.

## Biology is not behavior—or is it?

The FDA-NIH Biomarkers Definitions Working Group defined biomarkers as “a characteristic that is objectively quantified and assessed as an indicator of normal biological functions, pathological states, or pharmacological responses to therapeutic interventions” (31) to deliberately distinguish these from clinical outcome assessments (COAs). The distinction seems clear in principle: biomarkers reflect an objective biological property of the measurement itself, while COAs—which the FDA further subdivides into patient-reported outcome (PRO), observer-reported outcome (ObsRO), clinician-reported outcome (ClinRO), and performance outcome (PerfO) measures—describe “how a patient feels, functions, or survives” (32). Yet this taxonomy is more complex than it first appears. ClinRO measures can incorporate biologically anchored clinical findings—for example, stroke, myocardial infarction confirmed by electrocardiography (ECG) and creatine phosphokinase (CPK) blood test results), and PerfO measures—such as timed gait speed tests or word recall tasks—overlap substantially with measures sometimes presented as digital biomarkers (32). The biomarker/COA boundary turns not on a simple biology-vs.-behavior opposition, but on whether the measurement itself is *derived from* a biological process or instead captures a behavioral output used to *predict* biological or clinical states.

Yet this clarity dissolves under scrutiny. Behavior emerges from biological systems. When someone walks, neural circuits fire, neurotransmitters cross synapses, muscles contract, metabolic processes fuel activity. Behavior is the output of biology. So why isn’t behavioral measurement automatically biological measurement?

The answer lies not in whether behavior is biological—it obviously is—but in what information the measurement provides. Traditional biomarkers derive clinical utility from revealing biological processes that cannot be observed directly (12). HbA1c matters not merely because it correlates with diabetes outcomes, though it does, but because it reflects a specific biochemical process: the non-enzymatic glycation of hemoglobin in the presence of chronically elevated glucose (33, 34). This measurement provides information about underlying metabolic exposure that cannot be inferred reliably from symptoms or clinical observation alone.

Troponin is diagnostic for myocardial infarction not simply because elevated concentrations associate with heart attacks, but because cardiac troponins are released into the circulation following injury and necrosis of cardiac myocytes (35, 36). Measuring troponin therefore reveals a specific biological event—cellular injury and death—that becomes detectable in blood before, and sometimes in the absence of, definitive clinical symptoms (36).

The biological grounding is not incidental; it is what enables these biomarkers to work when symptoms are ambiguous, to detect disease before clinical manifestation, to distinguish different pathologies producing similar symptoms. The mechanistic connection between measure and biology is what makes them useful.

We should be careful not to oversimplify the biology–behavior distinction. Biological measures are themselves context-dependent and require careful interpretation. Cortisol provides a useful example: often described as a “stress biomarker,” cortisol levels vary substantially between individuals, follow circadian rhythms, and respond to numerous psychophysiological stimuli. A single cortisol measurement may provide less insight into an individual’s stress state than repeated self-report or behavioral observation. The distinction we propose is not that biological measures are inherently superior or more “objective,” but rather that they provide different types of information—direct measurement of a biological substrate vs. behavioral manifestation of a state that may have multiple biological and environmental determinants. Both can be context-dependent; both require careful interpretation; both can be valuable depending on the clinical question.

This suggests a criterion aligned with usage across the broader biomarker field (29, 30): the “bio-” in “biomarker” refers to a property of the *measurement itself*—the marker must be derived from biology—rather than merely to the nature of the outcome being predicted. A biomarker is not simply a “marker of biology” but a biologically derived marker. A behavioral correlate of a biological state, however statistically robust, does not qualify as a biomarker under this restrictive interpretation unless the measurement itself is grounded in a biological process. The question is not whether behavior is biological, but whether measuring behavior tells us about biology beyond what the behavior itself communicates.

## Three kinds of digital measures

We note that “digital” in this context refers to the mode of data acquisition and processing rather than defining a fundamentally different category of biomarker. Continuous glucose monitoring, photoplethysmography, and emerging spectroscopic measurements of molecular markers are all “digital” in their implementation. The meaningful distinction is therefore not digital vs. non-digital, but whether the measure directly assesses a biological variable (molecular or physiological), attempts to infer biological substrates from behavioral signals, or correlates behaviors with outcomes without biological intermediation. The “digital” modifier simply indicates that contemporary sensor and computational technologies enable these measurements. It does not alter the fundamental question of what, at a biological level, is actually being measured.

**Figure 1:**
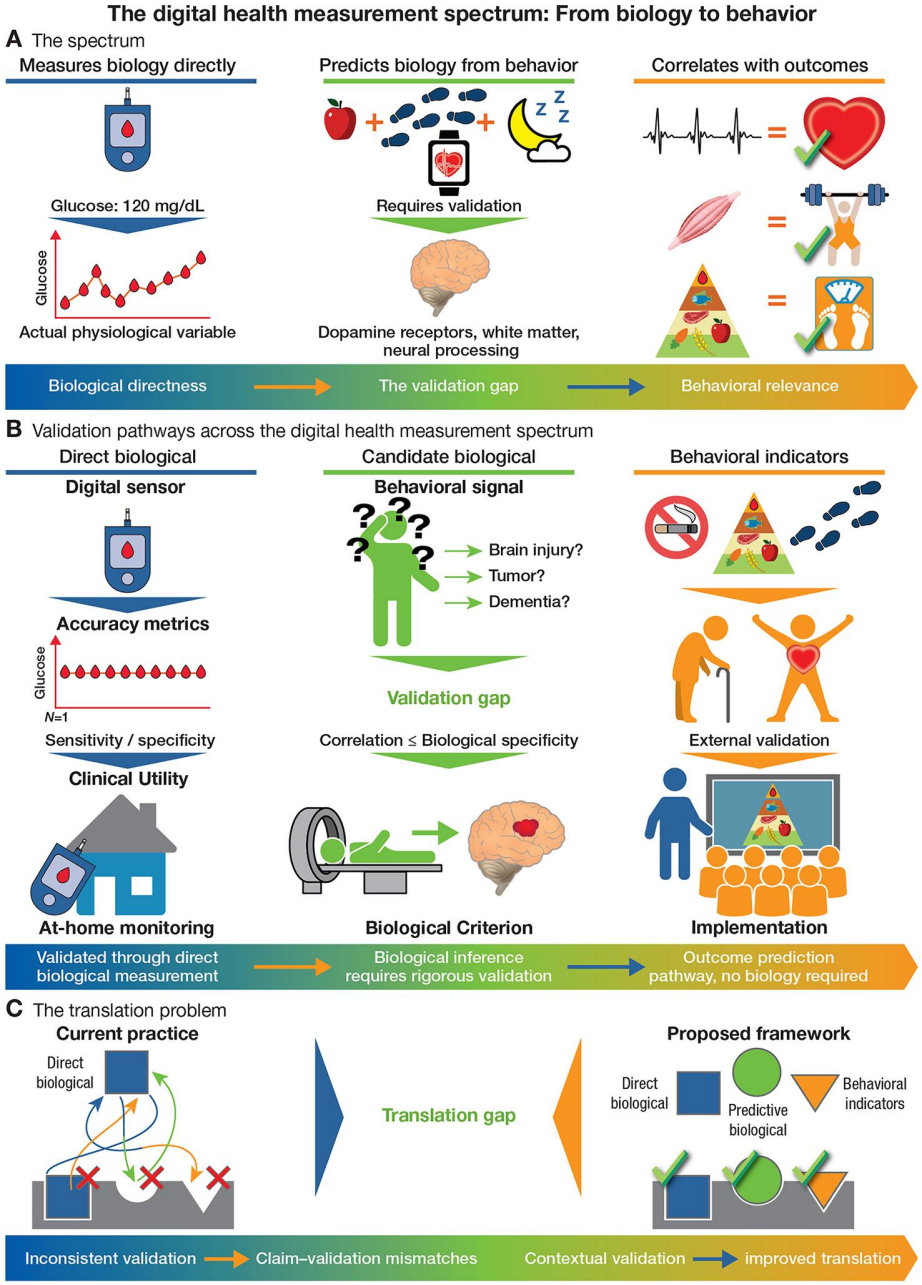
**The digital health measurement spectrum and category-specific validation pathways.** **(A)** Digital health measures span a spectrum from *direct biological* measurements (*left*, blue), through *candidate biological* measures inferred from behavior (*center*, green), to *behavioral indicators* that correlate with health outcomes without established biological mechanism (*right*, orange). Direct biological measures—such as continuous glucose monitoring—sense an actual physiological variable, providing a reading that maps onto an established biological quantity. Candidate biological measures combine behavioral signals (e.g., diet, activity, sleep) with the claim that these signals reflect specific biological substrates (e.g., dopamine receptor density, white matter integrity, neural processing speed); this claim requires validation before biomarker status is conferred. Behavioral indicators correlate passively sensed behaviors with clinical outcomes (e.g., ECG waveform predicting cardiovascular fitness, dietary pattern predicting weight) without requiring a mechanistic biological account. Moving rightward along the spectrum increases behavioral relevance while widening the validation gap in biological specificity. **(B)** Validation pathways differ by measurement type. Direct biological measures are validated against biological reference standards using accuracy metrics (sensitivity/specificity, limits of agreement). Candidate biological measures face a central *validation gap*: behavioral signals must be shown to correlate with biological criterion measures (e.g., neuroimaging, fluid biomarkers) before biological inference is warranted; correlation alone does not establish biological specificity. Behavioral indicators bypass biological validation entirely, instead requiring external outcome validation and demonstration of implementation performance in real clinical settings. **(C)** Under current practice (*left*), validation strategies are mismatched to measurement types—direct biological, candidate biological, and behavioral measures are validated interchangeably, producing systematic claim–validation mismatches and a persistent translation gap. The proposed framework (*right*) assigns category-specific validation pathways to each measurement type, aligning evidence standards with what each measure asserts and thereby ensuring that the evidence required for clinical adoption is appropriate to the measurement type and sufficient to support the decisions it is intended to inform.

Seen through this lens, digital health measures fall along a spectrum from direct biological measurement to pure behavioral correlation (Figure 1; Table 1). At one end sit measures that are simply digital instantiations of established biological assessments. Continuous glucose monitoring measures interstitial glucose via the same enzymatic reactions as laboratory assays, just continuously and remotely (23, 24). Photoplethysmography captures blood volume changes from which heart rate can be derived (37, 38), providing the same physiological variable as electrocardiography through different sensing modality. These measures are biological measurements that happen to use digital technology. They inherit validation standards from their non-digital counterparts: agreement with gold standard methods, accuracy specifications, reproducibility requirements.

**Table 1:** Validation requirements by measurement type.

Category	What it measures	Validation needed	Success criteria	Examples
Direct biological markers	Physiological variables via digital sensors	Technical accuracy against gold-standard biological measurements	High correlation with reference method; low bias; acceptable limits of agreement	Continuous glucose monitoring; wearable ECG; photoplethysmography-derived heart rate
Candidate biological markers	Behavioral signals claiming to reflect specific biological substrates	Correlation with biological criterion measures (imaging, fluid biomarkers, neural recordings)	Moderate-to-high correlation with biological substrate; mechanistic validation; information beyond clinical observation	Voice features validated against dopamine imaging; gait patterns correlated with white matter integrity; keystroke dynamics linked to neural processing
Behavioral indicators	Behaviors correlating with clinical outcome assessments without established biological mechanism	Prospective outcome prediction; incremental validity beyond self-report	Predicts clinical events; improves on existing assessment methods; generalizes across populations	GPS mobility and depression; smartphone usage and anxiety; social media activity and mood episodes

The translation question for these measures is primarily technical: is the digital measurement sufficiently accurate for clinical use? The Apple Watch ECG achieved FDA clearance not by proving atrial fibrillation is detectable, but by demonstrating their specific device detects it with sufficient sensitivity and specificity compared to 12-lead ECG (39, 40). The biological grounding was never in question; only the measurement quality required validation.

At the opposite end sit measures capturing behaviors that correlate with clinical states without established biological mechanism. GPS mobility patterns associate with depression severity (4, 5) and have increasingly been proposed as candidate clinical trial endpoints (41, 42). Indeed, people who are depressed move less, visit fewer locations, show reduced location diversity (25–27). This association is real, replicable, and potentially useful. But it reveals nothing about depression’s biological substrates. Reduced mobility might reflect depression’s motivational symptoms, or social withdrawal, or unemployment, or transportation access, or weather, or any combination of contextual factors. The measurement captures behavioral manifestation, not biological cause.

Such behavioral correlates can be valuable. They may predict clinical outcome assessments. They may trigger interventions. They may monitor symptom severity. But their validation requirements differ from biological markers. They need not demonstrate biological mechanism; they must prove predictive validity in relevant populations. They need not reveal hidden pathology; they must improve on existing assessment methods. They are outcome measures captured passively rather than biological markers sensed digitally.

For longitudinal monitoring applications—particularly in slowly progressive conditions like dementia—the framework accommodates trajectory-based approaches. A measure tracking deviation from an individual’s baseline gait pattern over months or years may serve as a valuable clinical indicator whether or not it validates against cross-sectional biological substrates. The key distinction remains: if the measure claims to reflect specific biological changes (e.g., white matter deterioration, dopaminergic loss), biological criterion validation is required. If it claims to detect clinically meaningful functional decline regardless of mechanism, outcome prediction validation is appropriate. Both purposes are legitimate; the framework simply requires clarity about which claim is being made.

Between these extremes lies the most conceptually interesting category: measures that capture behavior but claim to reflect underlying biology. These are candidate biomarkers—measures aspiring to biological derivation whose status remains contingent on biological criterion validation rather than already conferred by it. Voice analysis detecting Parkinson’s disease (43, 44). Keystroke dynamics predicting cognitive decline (45–47). Gait variability monitoring neurodegeneration or detecting early functional changes (48–50). These measures observe behavioral outputs—speech patterns, typing rhythms, walking dynamics—but argue these behaviors are tightly coupled to specific biological substrates: dopaminergic function, processing speed, cerebellar integrity.

The argument is plausible. Vocal production requires precise motor control dependent on basal ganglia function, which is disrupted by dopaminergic loss in Parkinson’s disease (51, 52). Similarly, typing speed and error patterns plausibly depend on processing speed and executive function linked to frontal-cortical integrity (53). Gait control involves multiple interacting neural systems whose dysfunction produces characteristic, measurable patterns.

But plausibility is not proof. The critical question is whether these behavioral measures provide information about biology beyond what clinical observation already reveals. Does voice analysis detect dopaminergic dysfunction before clinical symptoms emerge? Does it distinguish Parkinson’s from other conditions affecting motor control? Does it track biological disease progression independently of symptom severity?

Without validation against biological criterion measures—imaging, fluid biomarkers, direct neural recordings—we cannot know whether these measures capture biology or merely behavioral symptoms. The same gait variability might reflect cerebellar damage, vestibular dysfunction, peripheral neuropathy, muscle weakness, joint pain, fear of falling, or simple inattention. For applications targeting diagnosis or biological inference, demonstrating specific and independently verifiable relationships to underlying biological substrates is essential. For applications targeting longitudinal monitoring or early functional decline detection, rigorous outcome prediction validation across time and populations may be more appropriate. The framework clarifies which validation pathway best matches the scientific and clinical purpose of the application.

## Why the distinction matters

This conceptual framework is not taxonomic pedantry. It has immediate practical consequences for how research should be designed, evaluated, and translated.

Consider validation requirements. A measure claiming to directly assess a biological variable must prove accuracy against gold standard measurement of that variable. Continuous glucose monitoring required demonstration that interstitial glucose correlates tightly with laboratory blood glucose (23, 24). Photoplethysmography-derived heart rate required agreement with ECG-measured heart rate (13–15). The validation question is technical: does the digital measurement agree with the established biological measurement?

A measure claiming to predict biological substrates from behavioral signals faces a different validation burden. It must first demonstrate correlation with biological criterion measures. Voice features must correlate with dopamine imaging or autopsy-confirmed pathology. Gait patterns claiming to reflect white matter integrity must associate with quantified white matter disease or cerebellar volume. However, gait measures designed for longitudinal monitoring of functional decline need not validate against biological substrates if they make no claim to biological specificity. Only measures claiming biological grounding require biological validation before biomarker status can be asserted.

Without this biological validation, candidate measures remain behavioral indicators. They may still be useful—perhaps more useful than biological markers for certain applications. They may predict outcomes, monitor symptoms, trigger interventions. But they should be evaluated as outcome prediction methods, not biological markers. They need prospective validation against clinical events, not correlation with biological substrates.

The validation burden differs, and conflating these categories enables weak studies to claim strong conclusions. A study showing GPS mobility correlates with self-reported depression has not validated a biological marker. It has shown behavioral correlation with subjective symptoms—valuable information, but not biological measurement. Treating it as biomarker validation obscures what evidence remains missing.

We acknowledge that reference standards themselves can be layered, and validation pathways are not always linear. In myocardial infarction, ECG reflects physiological change, troponin reflects biochemical injury, and clinical diagnosis integrates both alongside presentation and outcomes. A digital method claiming to detect elevated troponin non-invasively should validate primarily against laboratory troponin measurement—the biological substrate being claimed—with concordance to ECG findings and clinical diagnosis serving as supporting evidence. The framework’s principle is that the validation standard should match the biological claim being made. If claiming to measure a specific biological variable, validate against direct measurement of that variable; if claiming to predict a clinical syndrome, validate against clinical diagnosis; if claiming to forecast outcomes, validate against outcomes. In practice, comprehensive validation may require multiple reference standards in a logical sequence, with the primary validation target determined by the measure’s central claim.

The distinction also shapes technical challenges differently. Device heterogeneity presents fundamentally different problems across measurement types. For direct biological measurements, different devices sensing the same physiological signal should converge after calibration. Systematic differences between photoplethysmography devices measuring heart rate reflect sensor quality, algorithm differences, or placement effects—technical problems requiring engineering solutions.

For behavioral correlates, device effects confound with user demographics and behavioral possibilities. iPhone vs. Android users differ in income, age, education. iOS vs. Android app ecosystems create different behavioral repertoires. “Device effects” are not merely measurement error but reflect different populations engaging in different behaviors through different platforms. Cross-device validation may be meaningless because devices do not measure equivalent constructs.

Similarly, context-dependence manifests differently. Biological measures are modulated by context in predictable ways amenable to modeling. Cortisol follows circadian rhythms and responds to stress; measuring at standardized times or modeling temporal patterns enables biological inference despite contextual variation.

Behavioral indicators are often inseparable from context. GPS mobility reflects employment status, caregiving responsibilities, weather, transportation access, and pandemic restrictions as much as mental state. Attempting to “control” for these factors to isolate depression-specific mobility changes may eliminate the signal entirely. The behavioral indicator captures lifestyle changes that accompany, cause, or result from depression. Behavioral and contextual factors are the variable of interest, not confounds to be removed.

This is precisely why validation frameworks must match what a measure claims to provide. As summarized in Table 2, mismatches between claims and validation standards systematically produce measures that appear statistically successful yet fail to translate clinically.

**Table 2:** Common claim–validation mismatches in digital biomarker research and their translational consequences.

Claim being made	Required reference standard	Common (incorrect) validation used	Consequence of mismatch
“Digital sensor measures a biological variable”	Gold-standard biological measurement of the same variable (e.g., ECG for heart rate; lab glucose for CGM)	Correlation with symptoms, self-report, or diagnostic labels	Apparent accuracy without biological validity; device appears useful but fails regulatory and clinical scrutiny
“Behavioral signal reflects a specific biological substrate”	Direct biological criterion measure (e.g., PET/SPECT imaging, neuroimaging, biofluid biomarkers, neural recordings)	Association with clinical diagnosis or symptom severity	Biological claims remain unsubstantiated; measure cannot distinguish mechanism from behavioral manifestation
“Digital measure detects disease earlier than clinical assessment”	Longitudinal biological change preceding symptom onset	Cross-sectional group classification (case–control accuracy)	Illusion of early detection; temporal ordering remains untested, limiting clinical trust
“Behavioral measure functions as a biomarker”	Demonstrated linkage to biological pathology beyond observable behavior	Prediction of self-reported outcomes or scale scores	Measure is misclassified; appropriate outcome-prediction utility is obscured by biomarker framing
“Algorithm generalizes across devices and populations”	Validation across heterogeneous devices with biological equivalence preserved	Performance replication within a single device ecosystem	Hidden population and context confounds; failures emerge at deployment

## The success stories are biological

Examining which digital measures have achieved clinical translation reveals a pattern. The successes are direct biological measurements.

Continuous glucose monitoring has transformed diabetes management (23, 24). These devices directly measure glucose concentration through enzymatic reactions identical in principle to laboratory assays. They required validation against blood glucose measurements, accuracy specifications, and demonstration of clinical utility when used to guide insulin dosing. But the biological grounding was never in question—only technical execution required proof.

The Apple Watch ECG received FDA clearance for atrial fibrillation detection (39, 40). Again, this is direct measurement of cardiac electrical activity, the same biological signal captured by traditional ECG through different sensing modality. Validation required demonstrating sufficient sensitivity and specificity compared to 12-lead ECG in relevant populations. Technical validation, not biological justification.

These examples succeeded because their path to clinical use was clear. They inherited validation frameworks from their non-digital counterparts. Regulatory agencies knew how to evaluate them. Clinicians understood what they measured. The biological grounding was transparent, making clinical utility assessable.

In contrast, measures claiming to infer biology from behavior remain largely research methods. Voice analysis for Parkinson’s disease shows promise but lacks the biological validation that would enable clinical adoption. Keystroke dynamics for cognitive assessment correlate with neuropsychological tests but have not been validated against neuroimaging or biofluid markers confirming they capture neural integrity rather than behavioral compensation. Without this biological grounding, clinical translation stalls regardless of statistical performance.

The pattern suggests that direct biological measurement, even using novel technologies, follows clearer paths to clinical adoption than behavioral inference, even when statistically robust. This is not because behavioral measures cannot be useful—they can—but because their utility requires different evidence and serves different functions than biological markers.

## The path through clarity

The digital health field stands at a critical juncture. Two decades of research have demonstrated technical feasibility and revealed associations between digital measures and clinical conditions (21, 22). Investment continues growing. Regulatory frameworks are developing. The question is whether this momentum translates into clinical impact or dissipates into another cycle of promising research without practical outcomes.

We propose that progress requires conceptual clarity before technical advancement. The field must decide what it is measuring and hold itself to standards appropriate for those measurements. This does not mean abandoning behavioral research or restricting innovation. It means being explicit about measurement types and honest about what evidence each requires.

For measures claiming direct biological assessment, the path is relatively clear: prove accuracy against established biological measurements, demonstrate clinical utility, secure regulatory approval. These are technical challenges with established precedents. Success requires rigorous execution but follows known pathways.

For measures claiming to predict biology from behavior, the requirement is biological validation before clinical translation. Show that behavioral signals correlate with biological criterion measures. Demonstrate mechanistic links between observed behaviors and biological substrates. Prove that digital measures provide information about biology beyond what clinical observation reveals. Only then can behavioral measures become biological markers.

For measures that correlate behaviors with outcomes without biological grounding, the appropriate framework is outcome prediction, not biological measurement. These measures should be evaluated on their ability to forecast clinical events, complement clinical assessment, and improve patient care. They may be valuable without being biomarkers. Calling them biomarkers does not strengthen their claims; it muddles their evaluation.

This conceptual discipline enables rather than constrains progress. It clarifies validation requirements, preventing wasted effort pursuing wrong evidentiary standards. It enables meaningful synthesis by grouping compatible measures. It provides clear regulatory pathways by matching measures to appropriate evaluation frameworks. It strengthens research by aligning methods with measurement types.

The alternative is continued confusion: studies applying incompatible validation standards, meta-analyses pooling incommensurable measures, clinical skepticism dismissing legitimate innovations alongside unsupported claims. Terminological promiscuity feels permissive but actually impedes progress by obscuring what must be proven and preventing appropriate evaluation. Worse, it creates a perverse equilibrium in which ever more papers can be published, grants can be justified, and products can be pitched on the strength of “biomarker” branding, even as the field quietly loses the shared criteria needed to distinguish measurement from prediction, correlation from mechanism, and genuine clinical readiness from statistical novelty.

## Embracing specificity

Some will worry that conceptual boundaries stifle innovation. If we restrict “biomarker” terminology to biologically grounded measures, does this devalue behavioral research? Does it close doors to novel approaches?

The opposite proves true. Clarity enables innovation by directing research toward appropriate goals. Behavioral correlates can be enormously valuable for screening, monitoring, and intervention triggering. Freeing them from inappropriate biomarker validation requirements lets them be evaluated on their actual merits: prediction accuracy, clinical utility, practical feasibility. Forcing them into biomarker frameworks creates barriers they need not overcome.

Consider predictive models for hospital readmission. These incorporate demographics, prior utilization, comorbidities, functional status, and social determinants. They predict important outcomes without claiming to measure biological processes. Their utility derives from outcome prediction, not biological insight. We do not demand they validate against molecular markers or reveal disease mechanisms. We evaluate them on discrimination, calibration, and decision curve analysis. They serve clinical purposes without being biomarkers.

Digital behavioral measures could follow similar paths. GPS mobility predicting psychiatric relapse. Smartphone usage forecasting depression recurrence. Social media activity flagging suicide risk. These could be evaluated as prediction methods serving specific clinical functions, assessed on their ability to improve outcomes when integrated into care pathways. The validation burden would be substantial but appropriate: prospective studies, external validation, decision impact analysis, implementation research.

Freeing these measures from biological validation requirements does not diminish their potential value but rather focuses evaluation on what they actually provide: behavioral information with potential clinical utility. This is not a lesser goal but a different one, meriting specific frameworks and rigorous standards.

Meanwhile, measures attempting to infer biology from behavior face a clearer research agenda: demonstrate biological grounding before claiming biomarker status. Conduct the studies linking behavioral signals to biological substrates. Show that voice features correlate with dopamine imaging findings. Prove that gait patterns track white matter integrity. Validate that keystroke dynamics reflect neural processing capacity beyond behavioral task performance.

This research is challenging and resource-intensive. It requires combining digital measurement with expensive biological assays: neuroimaging, PET scanning, cerebrospinal fluid sampling, genetic sequencing. It demands large samples, longitudinal designs, multi-site collaborations. But this is what biological validation requires. Avoiding this work while claiming biomarker status creates illusion of progress while the translational gap persists.

## Anticipating objections

The proposed framework will inevitably face objections. Addressing them clarifies rather than weakens the argument.

**Objection 1: Biological and behavioral systems are deeply intertwined—strict boundaries are artificial.** This objection is correct about entanglement but wrong about implications. Yes, all behavior emerges from biology. But the question is not whether behavior is biological, but what information measurement provides. Blood pressure emerged from cardiovascular biology yet became a biomarker because measuring it revealed information about cardiac function and vascular resistance not apparent from observing the person. The framework does not deny connections; it asks what each measurement tells us that we could not know otherwise.

**Objection 2: Strict categorization might prematurely dismiss novel approaches.** The framework does not dismiss innovations but clarifies their validation requirements. A novel measure claiming to predict dopaminergic function from voice should validate against dopamine imaging—challenging but not dismissive. A novel behavioral predictor of depression relapse should validate through outcome prediction—different validation, not lesser value. The framework prevents premature claims while preserving space for ambitious research. What it eliminates is claiming biological insight without biological validation.

**Objection 3: Many established biomarkers began as behavioral observations before mechanisms were understood.** Historical examples like blood pressure support rather than contradict the framework. Blood pressure began as a correlate of cardiovascular disease but achieved biomarker status through mechanistic understanding linking pressure to pathophysiology. The framework allows measures to transition categories as evidence accumulates. What began as behavioral correlation becomes a biological marker once mechanism is demonstrated and validated. The framework describes current evidence status, not permanent classification.

**Objection 4: The boundaries are unclear and determining sufficient biological validation is subjective.** Boundary cases will exist, but this does not invalidate the framework—it makes explicit what requires judgment. A measure showing weak correlation with one biological criterion study remains a behavioral correlate. A measure showing robust, replicated correlations with multiple biological substrates across independent samples gains biological grounding. The framework provides principles for evaluation even when specific cases require nuanced judgment. Ambiguity at boundaries does not justify abandoning the distinction between direct measurement, validated prediction, and behavioral correlation.

**Objection 5: Biological validation is prohibitively expensive—this framework privileges well-funded research.** Resource intensity is real but does not justify lowering standards. If a measure cannot afford biological validation, it should not claim biological insight. It can still pursue outcome prediction validation, potentially providing equal or greater clinical value. The framework reveals where investment is needed, potentially attracting funding for essential validation studies. Claiming biological grounding without biological validation does not solve the resource problem; it creates illusions of progress while translation fails.

For early-stage research with limited resources, the framework provides a clear path: present the measure as a candidate biomarker requiring biological validation rather than claiming biomarker status. Report associations with behavioral or clinical outcomes while explicitly noting that biological grounding remains to be established. Position the work as hypothesis-generating, identifying promising signals that merit investment in biological validation studies. This honest positioning does not diminish the contribution—it accurately represents the current state of evidence and identifies the critical next steps. Investigators can also pursue collaborative approaches, partnering with groups that have access to biological criterion measures, or focus on conditions where less expensive biological validation is feasible. The framework does not demand that every research group conduct resource-intensive validation independently, but it does require that biological claims be supported by biological evidence before biomarker status is asserted.

These objections clarify that the framework is not about restricting innovation or creating rigid hierarchies. It is about matching claims to evidence and validation standards to measurement types. Measures can be valuable without being biomarkers. Claims can be ambitious while remaining honest about what has been proven. The framework provides structure for evaluation without prescribing which measures matter most.

## Learning from heartbeats

Perhaps an analogy helps. Consider the humble heartbeat. We can measure it many ways: finger on the wrist feeling pulse, stethoscope to chest hearing sounds, ECG recording electrical activity, photoplethysmography sensing blood volume changes, ultrasound visualizing chamber motion (54, 55). Each method captures different physical manifestations of the same biological phenomenon.

The pulse at the wrist is easily accessible but provides limited information: rate and rhythm, gross regularity. The stethoscope adds sound quality revealing valve function. The ECG reveals electrical conduction, detecting arrhythmias invisible to pulse palpation. Echocardiography shows structural and functional abnormalities (56). Each measurement provides different biological information despite all reflecting cardiac activity.

Now imagine someone proposes measuring heartbeat indirectly: monitoring how often someone checks their pulse, or analyzing diary entries mentioning cardiovascular symptoms, or tracking pharmacy refills for cardiac medications. These behavioral correlates might predict cardiac events or disease burden (57). The correlations could be statistically robust.

But these behavioral measures would not replace ECG or echocardiography for biological assessment. They provide different kinds of information, serving different purposes of equal clinical value, and operating under different validation standards. The behavioral measures might help screen populations, flag risk, or monitor adherence and symptom burden over time. The biological measures, by contrast, diagnose pathology, localize mechanism, and guide treatment decisions. Both have value, but conflating them would blur what each actually reveals and could reveal—and why that difference matters. The distinction is about matching validation to purpose rather than ranking importance.

Digital health measures present similar situations. Some directly sense biological signals using novel technologies—replacing the finger on the wrist with the optical sensor in a watch. Others infer biology from behavioral proxies—tracking activity patterns rather than measuring cardiac rhythm. Still others correlate behaviors with outcomes without biological intermediaries—monitoring medication refills rather than heart function. All may contribute to clinical care. But they contribute differently, require different validation, and merit different evaluation. Clarity about these differences enables each measurement type to realize its potential rather than forcing all into a single framework where some inevitably fail by inappropriate standards.

## The question that guides research

Every research program faces a fundamental question shaping design, methods, and interpretation. For digital biomarker research, that question should be: What kind of information does this measure provide (31)?

If the measure directly assesses a biological variable, research should focus on measurement validation: accuracy, precision, reliability, agreement with gold standards. Success means technical performance meeting clinical requirements. Translation follows established regulatory pathways with clear acceptance criteria (21, 22).

If the measure claims to predict biological substrates from behavioral signals, research must establish biological grounding. What biological variables does the behavior reflect? What evidence links digital signals to those substrates? What studies demonstrate correlation with biological criterion measures? Success means proving the behavioral measurement reveals biology beyond what behavioral observation provides.

If the measure correlates behaviors with outcomes without biological mechanism, research should address prediction performance and clinical utility. Does it forecast relevant outcomes? Does it improve on existing methods? Does it change clinical decisions? Does it enhance patient care? Success means demonstrating value for specific clinical applications through appropriate validation frameworks.

Framing research around these questions enables appropriate study design and realistic evaluation. It prevents studies generating statistically significant findings that do not address the essential evidence their claims require. It guides resource allocation toward the validation each measure needs rather than the validation convenient to conduct.

Most importantly, it makes research cumulative. When studies address the right questions, findings build on each other. Validation frameworks become established. Standards emerge. Translation pathways clarify. The field develops shared understanding of what evidence constitutes progress.

Without this clarity, research remains fragmented. Each study invents its own validation criteria. Meta-analyses cannot synthesize findings from incompatible frameworks. Translation stalls because no one knows what constitutes sufficient evidence. The literature grows; understanding stagnates.

## An invitation to precision

This essay has traced boundaries between biological measurement and behavioral correlation, arguing that conceptual clarity enables progress that terminological promiscuity prevents. The argument may seem constraining, imposing categories and demanding specificity in a field celebrating innovation and interdisciplinarity.

But precision is not constraint. Precision is the foundation enabling ambitious research to achieve meaningful outcomes. When we know what we are measuring, we can design studies providing appropriate evidence. When we apply validation standards matching measurement types, we can interpret findings accurately. When we synthesize compatible evidence, we can build cumulative knowledge. When we chart clear paths to translation, we can realize clinical impact.

The digital health field has generated remarkable innovations: sensors worn comfortably for continuous monitoring, algorithms extracting patterns from streams of data, platforms enabling research at unprecedented scale. These technical achievements deserve rigorous science that does them justice. That science requires conceptual foundations as sophisticated as its technologies.

We propose a simple discipline: be explicit about what you are measuring and hold yourself to standards appropriate for that measurement. If claiming biological assessment, prove biological grounding. If predicting biology from behavior, validate against biological criteria. If correlating behavior with outcomes, demonstrate prediction performance. Do not claim biological measurement while providing behavioral correlation. Do not validate against behavioral outcomes while claiming biological insight.

This discipline does not divide the field into rigid categories. Measures may span boundaries or shift categories as evidence accumulates. Behavioral indicators may gain biological grounding through validation studies. Biological measurements may reveal behavioral insights. The boundaries are not barriers but guides for appropriate research design and honest evaluation.

The ultimate goal remains the same: translating digital technologies into technology that improves clinical care. Achieving this requires innovations in sensing, algorithms, and implementation—but also in conceptual clarity about what we are measuring and what evidence our claims require. Technical sophistication without conceptual precision produces research that looks like progress but leads nowhere clinically.

The field can continue its current trajectory: expanding publication counts, growing commercial investment, persistent translational failure. Or it can embrace the discipline of clarity: explicit about measurement types, rigorous about appropriate validation, honest about remaining uncertainties. This path is harder—requiring difficult validation studies, acknowledging limitations, resisting overstatement—but it is the path toward clinical impact that genuinely improves patient care.

The choice is ours. The time is now. The question is simple: What are we actually measuring? The answer shapes everything that follows.

## Data availability statement

The original contributions presented in the study are included in the article/Supplementary Material, further inquiries can be directed to the corresponding author.
